# Total body MRI, a guide to diagnosis in patients with osteo-articular pain and inflammation

**DOI:** 10.1186/1546-0096-12-S1-P164

**Published:** 2014-09-17

**Authors:** Lien De Somer, Carine Wouters, Steven Pans

**Affiliations:** 1 UZLEUVEN, Leuven, Belgium

## Introduction

CRMO is an autoinflammatory bone disorder, characterized by aseptic multifocal osteitis. MRI is classically used in measuring the extent and activity of the bone lesions and possible soft tissue involvement. Additionally, MRI may help to exclude other causes of osteoarticular inflammation.

## Objectives

To demonstrate the usefulness of whole body MRI in the differential diagnosis of recurrent bone pain with inflammation in children.

## Methods

Retrospective study of pediatric cases with bone pain and inflammation. Comprehensive imaging study including whole body MRI.

## Results

Two patients were referred to our pediatric rheumatology outpatient clinic with the presumed diagnosis of CRMO. Since several months, they experienced episodes of articular pain and joint swelling (patient 1) and recurrent back pain and fatigue (patient 2). In the first patient initial blood evaluation showed mildly increased inflammatory parameters (CRP 75 mg/L) with a mild microcytic anemia (Hb 11 g/dL) and thrombocytosis (557x10e6/µl) with a normal leukocyte count. Conventional X-ray showed no abnormalities. MRI of both ankles confirmed arthritis/synovitis with presence of bone edema in ankle and tarsal bones. Initial treatment with NSAIDs was successful. In patient 2 additional investigations showed also increased inflammatory parameters (CRP 136 mg/L) with a mild anemia, slightly increased thrombocytosis and normal leukocyte count. Initial MRI of the spine showed mildly intense signaling of different vertebrae on STIR images. Bone scintigraphy demonstrated multifocal hypercaptation at the right skull, coracoied bone and multiple vertebrae. Additional bone marrow puncture was normal. NSAIDs were started systematically with success.

In patient 1, pain returned. PET-CT showed increased metabolism at multiple skeleton foci. Additional bone marrow puncture in the posterior iliac crest returned normal with no arguments for a lymphoproliferative disorder. Persistent and worsening of clinical symptoms prompted us to perform whole body MRI which showed multiple, symmetric T1-hypointens signals in the peripheral bones (lower legs, underarms, feet, knees and elbows). Guided by these findings, a targeted bone marrow puncture was performed in the tibia which showed a malignant lymphoproliferative disorder, namely a pre-B ALL.

Also in patient 2, pain returned and became progressive over a few weeks. A whole body MRI showed a progressive disease with intermediate signal intensity on STIR and hypointense signaling on T1-weighted images at different vertebrae and the sacral bone. A second bone marrow puncture at the crista returned to be negative. Guided by the MRI images a bone biopsy was performed in the sacral bone and was compatible with the diagnosis of an ALCL.

## Conclusion

CRMO remains a diagnosis of exclusion in patients with osteoarticular pain and inflammation. In case of persistent and/or progressive pain, the physician should keep a high index of suspicion for malignancy. Whole body MRI can precisely define the characteristics and extent of the bone lesions (aspect and localization) as well as help in choosing the optimal localization for bone marrow puncture and/or bone biopsy.

## Disclosure of interest

None declared.

